# German translation and validation of the Reporting of Clinical Adverse Events Scale (RoCAES-D)

**DOI:** 10.1186/s12913-020-05546-2

**Published:** 2020-07-25

**Authors:** Nicola Alexandra Litke, Michel Wensing, Antje Miksch, Katja Krug

**Affiliations:** Department of General Practice and Health Services Research, University Hospital Heidelberg, Im Neuenheimer Feld 130.3, 69120 Heidelberg, Germany

**Keywords:** Adverse events, Incident reporting, Patient safety, Risk management, Safety culture

## Abstract

**Background:**

Reporting of adverse events is an important aspect of patient safety management in hospitals, which may help to prevent future adverse events. Yet, only a small proportion of such events is actually reported in German hospitals. Therefore, it is crucial to evaluate attitudes of clinical staff towards reporting of adverse events. The aim of this study was to translate the Reporting of Clinical Adverse Events Scale (RoCAES) developed by Wilson, Bekker and Fylan (2008) and validate it in a sample of German-speaking health professionals.

**Methods:**

The questionnaire covers five factors (perceived blame, perceived criteria for identifying events that should be reported, perceptions of colleagues’ expectations, perceived benefits of reporting, and perceived clarity of reporting procedures) and was translated into German language according to translation guidelines. Within a cross-sectional study in a sample of 120 health professionals in German hospitals, internal consistency (omega) and construct validity (confirmatory factor analysis) of the German scale RoCAES-D was assessed.

**Results:**

The reliability was high (omega = 0.87) and the factor analysis showed a poor model fit (RMSEA: 0.074, χ^2^/df: 1.663, TLI: 0.690). Resulting from lower model fit of the original model (RMSEA: 0.082, χ^2^/df: 1.804, TLI: 0.606), one item was deleted due to low factor loadings and a low R^2^ (0.001), and two items were reallocated from the factor ‘perceived benefits’ to ‘perceived blame’.

**Conclusion:**

The successful translation and initial validation of the RoCAES-D might be a good starting point for further research. A cultural adaptation of the scale needs to be done to initiate a large-scale usage of the questionnaire.

## Background

Patient safety indicates the absence of adverse events (AE), which has received increased interest as it is endangered by the increasing complexity of healthcare, the simultaneously increasing economic pressure and the shortages in the healthcare work force [1]. An AE is defined as an unintended injury resulting from a medical intervention, regardless of the patient’s underlying medical condition [2, 3]. Prevention of AEs has been the focus of many improvement initiatives and studies across the world [4].

The occurrence of AEs varies between 3 and 17% of all hospital stays [1, 5, 6]. In Germany, AEs are expected to affect 5–10% of all patients in the inpatient sector, implying 950.000–1.900.000 AEs per year [3]. Up to 60% of these AEs are considered to be preventable, resulting in approximately 19.000 avoidable deaths per year in Germany [3, 6, 7].

To understand structures and procedures in health care that might lead to an AE, it is crucial to analyse them objectively assuming AEs being reported at first [8]. Hospital risk management, which is obligatory in German hospitals according to prevailing regulations of the Joint Federal Committee (gemeinsamer Bundesausschuss) since 2014, contains instruments to reporting of AEs and critical incidents in general [3, 9]. The critical incident reporting system (CIRS) is one of these instruments and comprises a platform where hospital employees can anonymously report AEs as well as other critical incidents that occur in the context of health care and increase the risk for AEs. These reports are then analysed by experts who give recommendations for the future avoidance of events of this kind [10].

However, there is a range of barriers that impede AE reporting. As possible reasons for underreporting of AEs, a lack of support from authorities or the person ‘causing’ the error, fear of consequences, lack of knowledge and high workload were named [11, 12]. There is strong evidence, that the number of unreported or unpublished AEs is much higher than the ones being reported and included in AE estimations leaving approximately up to 91% unreported [3, 13, 14].

Beneficial safety culture is necessary to achieve a supporting environment and thus encourage staff to report AEs [8]. In Germany, within the last 15 years the ways of dealing with errors and AEs has changed. From avoiding this topic up to then, to approaches that aim to analyse factors that contribute to the occurrence of errors and also include an organisational perspective [3]. However, still more than half of German hospitals are estimated to not ensure an optimally beneficial safety culture that includes the ability to learn from reported incidents [15]. To promote a supportive safety culture in general, it is necessary to understand attitudes and values of employees towards adverse event reporting [4].

Yet, there is no German-language questionnaire assessing health professional’s attitudes to AE within German health services.

The Reporting of Clinical Adverse Events Scale (RoCAES) was developed and validated in the UK by Wilson, Bekker and Fylan in 2008 and assesses five-domains of staff attitudes to AEs: perceived blame (six items); perceived criteria for identifying events that should be reported (six items); perceptions of colleagues’ expectations (six items); perceived benefits of reporting (five items); perceived clarity of reporting procedures (two items) [16]. It was translated and validated for use with health professionals in China [17], and may be a robust measure for use within a German healthcare system. The aim of this study was to translate this questionnaire into German and assess its reliability and validity for use to assess attitudes of health professionals to AEs in German hospitals.

## Methods

The questionnaire was translated into German language according to international translation guidelines. Psychometric properties of this translated version were then examined in a cross-sectional study in German hospitals.

### Translation process

The translation of the RoCAES is based on international recommendations [18–20], following the five steps described below:

- Forward translation: The instrument was translated from English into German by two independent translators whose mother tongue is German and that are fluent in English.
- Comparison of the two translated versions: Within step a), two translated versions of the questionnaire were produced. A consensus meeting was held to discuss discrepancies and ambiguities of these two versions and to generate the preliminary initial translated version.
- Blind back-translation: Two bilingual translators, whose mother tongue is English, were asked to independently translate the preliminary initial translated version back into the source language. For this step, translators with no initial knowledge of the instrument were chosen.
- Comparison of the two back-translated versions: Within a second consensus meeting, discrepancies in sentence structures, meaning or other uncertainties have been discussed and an English consensus version has been merged. In addition to this step, the back-translated version has been sent to the original author of the RoCAES who compared this version with her initial intentions of the original questionnaire to ensure conceptional equivalence.
- Pilot testing of the pre-final version: several independent experts were asked to review the German pre-final version of the questionnaire. Furthermore, five experts matching the inclusion criteria for the subsequent survey were involved in a cognitive debriefing evaluating instructions, items and response format clarity.

### Study population and data collection

For data collection, the RoCAES-D was transferred into an electronic version. The link to the online survey was sent to approximately 1300 staff members in 18 different institutions (14 hospitals, a medical school, a nursing school, a school for therapists and a germanwide network of professionals interested in incident reporting) step by step in spring 2019. Study population included all clinical staff members and students that work in the inpatient care sector and have direct patient contact (e.g. nurses, physicians, therapists, …). We covered this interprofessional perspective in the German version contrarily to the eligibility criteria of Wilson et al. and Sun et al. [16, 17], who addressed doctors and nurses only, as interprofessional teamwork plays an important role in patient safety and prevention of clinical incidents [21].

The hospitals cover various clinical departments such as neurology, orthopedy, rehabilitation clinics, geriatrics or internal medicine. Within the hospitals, the survey was sent to the management, quality management and staff councils via e-mail. They were asked to pass on the mail to their employees. In addition, we offered to send paper-based questionnaires to reach staff without access to their e-mail accounts. Because management staff is not included in the target group addressed by the questionnaire, an initial filter question excluding unsuitable test persons was added. Furthermore, the clinical staff was also asked to send the link to their colleagues, former fellow students or friends matching the eligibility criteria (snowball system).

The targeted sample size was estimated to be within the range of 100 to 250 in order to facilitate the planned confirmatory factor analysis (CFA) [19, 22, 23].

### Measures

The RoCAES consists of 25 items rated with a 4-point-likert scale from ‘1’ (strongly agree) to ‘4’ (strongly disagree). 16 items required reversed scoring. As agreeing to item 4 (‘reporting adverse events protects patients’) results in a positive attitude and item 3 (‘It is not my responsibility to report adverse events involving colleagues’) is formulated negatively, we assumed that the scoring guideline for these two items was switched in the original study. Contrary to the recommendation, we therefore reverse scored item 3 instead of item 4 for our analysis.

Since the questionnaire was sent in an online version and included mandatory fields, the answer option ‘not specified’ had to be inserted.

An introductory definition of AEs and three additional items introduce the main scale focusing on whether the participant has ever witnessed or been involved in an AE, if he ever reported an AE and how likely the participant is to report an AE in the future. Closing the questionnaire, demographic data was collected including profession, gender and previous duration of employment. The student employee status was not assessed separately. The full German questionnaire can be found in the Additional file 1.

### Data analysis

To assess reliability and validity of the RoCAES-D, only fully completed questionnaires were analysed. For further analysis, answers that chose ‘not specified’ were treated as missing data and have been imputed using the mean value of each item across participants [24]. Internal consistency was determined by calculating coefficient McDonald’s omega which can be used for multidimensional tests as a more suitable alternative to Cronbach’s alpha [25]. To find omega, a factor analysis is done by rotating the factors obliquely and performing a Schmid Leiman transformation. Cronbach’s alpha was assessed as well to provide better comparability and transparency. Alpha was calculated for the overall scale following the original authors and the Chinese validation paper [16, 17].

A CFA was selected for testing construct validity. For this, the factor model was based on data of original research by Wilson et al. and Xiao Sun et al. [16, 17] and was used to test its model fit for the empiric research data. Data analysis was done using R Version 3.5.1 and SPSS Version 25. In R we used the procedures ‘omega’ within the psych package and ‘cfa’ within the lavaan package.

The CFA was done using the original factor structure of the RoCAES. A one-factor model was used to compare model fit showing no relevant benefit for the existing five-factor model. For calculating reliability, the used coefficient McDonald’s omega estimates the general factor saturation of a questionnaire by doing an exploratory factor analysis (EFA) first. This exploratory approach was used, as well as residuals and covariances calculated within the CFA, to modify the existing factor model.

More precisely, items with a low factor loading (less than 0.25) and a low number of explained variance (R^2^ less than 0.1) were reallocated using the highest observed factor loadings in the exploratory analysis that was done to estimate omega.

## Results

The translation process did not reveal any noteworthy discrepancies or ambiguities. The pilot testing via cognitive debriefing has led to an adaption of two sentences, that had to be changed marginally due to incongruities in sentence structure. Observed associations triggered by the phrasing of the items agreed with each other and with the intention of the questionnaire. Together with the standardized introduction, and the written instructions, objectivity of application can be assumed as given for this scale.

Furthermore, data analysis is clearly defined by referring to a fixed scoring for each answer option. The objectivity of the evaluation is therefore also seen as given.

The psychometric testing of the pre-final German RoCAES-D was conducted in spring of 2019. A total of *n* = 120 health professionals in 26 hospitals completed the survey. Because the recruitment was conducted as a snowball system, response rate cannot be estimated accurately. The sample included heterogeneous professions and work experience. Sociodemographic characteristics of the sample are shown in Table 1.

**Table 1:** sociodemographic data (*n* = 120)

	N	%^**a**^
Gender		
male	22	18.3
female	96	80.0
not specified	2	1,7
Professions		
doctors	7	5.8
nurses	80	66.7
therapists^b^	26	21.7
others	6	5.0
not specified	1	0,8
Previous duration of employment in this institution		
< 1 year	10	8.3
1–2 years	17	14.2
2–4 years	27	22.5
5–10 years	23	19.2
> 10 years	41	34.2
not specified	2	1,7

^a^participants were given an additional option to officially not chose one of the listed answer options: ‘keine Angabe’, labelled as ‘not specified’ in this table

^b^therapists such as: speech and language therapists, physio therapists, occupational therapists, etc.

### Confirmatory factor analysis

For the CFA, as the answer options ‘not specified’ were treated as missing values, two items showed no missings. In all other items, missing values range from 0.8 to 16%. The two items with the highest missing rate include Item 11 (‘My colleagues expect me to report adverse events’, 16% missing values) and Item 13 (‘The procedures in this hospital are clear on what sort of adverse events should be reported’, 12.5% missing values). Other items showed missing rates lower than 7.5%.

Results of the initial CFA showed a poor model fit (Table 2) for the original structure in the German context. Using the one-factor model to compare model fit also showed no relevant benefit for the existing five-factor model. Therefore, the original model was modified as described previously. This modified model resulted in the lowest RMSEA*, SRMR*, and AIC* (Table 2). Hence, it is considered to be the one with the best model fit [19, 22].

**Table 2:** model fit of the existing model, a one-factor model and the modified model

Model	Chi-squared	Df	normed Chi-squared	RMSEA	SRMR	CFI/TLI	AIC
existing model	486.949	270	1.804	0.082 90% CI [0.070–0.093]	0.115	0.646/0.606	6778.084
one-factor model	535.334	275	1.947	0.089 90% CI [0.078–0.100]	0.103	0.575/0.536	6816.469
modified model	410.814	247	1.663	0.074 90% CI [0.061–0.087]	0.092	0.723/0.690	6409.801

*Df degrees of freedom, normed Chi-squared is calculated Chi-squared/df, *RMSEA* root mean square error of approximation, *SRMR* standardized root mean squared residual, *CFI* Bentler’s Comparative Fit Index, *TLI* Tucker-Lewis Index, *AIC* Akaike’s Information Criterion

Classifying the measures, the modified model showed a poor model fit for the German target group. Modifications in this model conclude the deletion of item 9 (‘I am not doing my job properly unless I report adverse events’) because of low factor loadings (0.024) and a low R^2^ (0.001). Item 1 (‘Reporting adverse events helps identify staff who need additional training’) and Item 24 (‘Reporting adverse events makes people accountable for their actions’) originally loaded on factor 4 (‘Perceived benefits of reporting’) and have now been relocated to factor 1 (‘Perceived blame’). Both items showed low factor loadings within the initial CFA and a correlation with other items within the EFA. Additionally, a content-related alignment lead to the described reallocation. Item 4 (‘Reporting adverse events protects patients’) also required a reallocation. However, a new assignment was not reasonable due to a bad model fit and a content-related match with the original factor 4 (perceived benefits). This resulting 24-item questionnaire can be seen in Table 3.

**Table 3:** factor loadings of the modified 24-item RoCAES-D

Items RoCAES-D	loading
Factor 1 Perceived Blame	
1. Reporting adverse events helps identify staff who need additional training	−0.332
5. Reporting adverse events lets others check up on me	0.433
7. The careers of staff who report adverse events suffer	0.724
12. Reporting adverse events creates problems for me	0.820
18. Reporting adverse events lets everyone know I have made a mistake	0.564
21. Reporting adverse events is a method through which to pinpoint blame	0.613
23. Reporting adverse events lets colleagues gossip about my involvement in the event	0.600
24. Reporting adverse events makes people accountable for their actions	−0.478
Factor 2 Perceived criteria for identifying events that should be reported	
2. Whether or not to report an adverse event depends on how many people are aware the error has taken place	0.421
3. It is not my responsibility to report adverse events involving colleagues	0.317
10. Minor adverse events should not be reported	0.484
14. Only uncommon adverse events should be reported	0.471
15. Writing in a patient’s notes that an adverse event has happened is just as good as filling in a separate reporting form	0.288
20. You should only report those adverse events where something can be learnt from them	0.583
Factor 3 Perceptions of colleague’s expectations	
6. As long as those around me learn from adverse events there is no need to report them	0.512
11. My colleagues expect me to report adverse events	0.257
19. I am not permitted to report adverse events	0.378
22. Adverse events can’t be prevented so there is no point in reporting them	0.573
25. Colleagues seem unconcerned when adverse events occur	0.320
Factor 4 Perceived benefits of reporting	
4. Reporting adverse events protects patients	0.239
16. Receiving encouragement from senior clinical staff encourages me to report adverse events	0.755
17. Having an Adverse Event Monitoring Unit based in the Hospital encourages staff to report errors	0.604
Factor 5 Perceived clarity of reporting procedures	
8.The procedures in this hospital are clear on how to report adverse events	0.804
13. The procedures in this hospital are clear on what sort of adverse events should be reported	0.652

### Reliability

For the translated pre-final version, omega was 0.85, implying a high factor saturation. In order to ensure better comparability, Cronbach’s alpha was also calculated and was 0.79. Both coefficients were again estimated for the modified 24-item RoCAES-D. Omega increased to 0.87, alpha also increased to 0.81 (Factor 1: 0.8; Factor 2: 0.53; Factor 3: 0.45; Factor 4: 0.53; Factor 5: 0.68).

## Discussion

The resulting RoCAES-D shows high reliability and a poor validity for the German target population. Cronbach’s alpha was somewhat lower than but still comparable with the English RoCAES (0.83) and the Chinese C-RoCAES (0.85) [16, 17]. However, coefficient omega showed a high reliability (0.87). The even poorer model fit of the one factor model (Table 2) in the RoCAES-D underlines the multidimensional structure of the scale [25].

The original factor structure did not have a good fit with the data. Reallocating items 1 and 24 may result from cultural differences between German and English health professions. The two items were originally associated with benefits of reporting and now correlate with perceived blame. As risk management and handling adverse events is not an official part of the professional education in German health professions [26, 27], staff is not inevitably habituated with reporting processes, implying a perceived blame when actually practiced. In contrast, being aware of risks of medical interventions and being familiar with reporting procedures is part of the English competence model of nurses [28], resulting in a more neutral and professional point of view perceiving benefits instead of blame and implying a higher rate of incident reporting [4]. The observed negative loadings of those two items (Table 3) might also support the cultural differences that have been identified throughout the reallocation. Furthermore, resulting from this different understanding of professional roles, German health care workers might not primarily associate their professional duties in health care with the responsibility of reporting adverse events, which can be seen in the CFA: item 9 (‘I am not doing my job properly unless I report adverse events’) showed particularly low factor loadings on any of the five factors and an average R^2^ of 0.001 and was therefore deleted for the RoCAES-D.

Due to the imputation of missing values, an impact on variance and therefore on the estimated psychometric properties leading to a miscalculation needed to be considered. Yet, the proportion of imputed values stayed relatively low with only two items higher than 7.5% (16% and 12,5%). In this order, no considerable impact is expected [29].

Besides the acceptable psychometric properties of the RoCAES-D, the usage of the scale in a multiprofessional target group has proven to be practical. This population differs from the original survey in England, which only included nurses and doctors. The German scale also addressed other health professions that are directly involved in daily patient care. In addition to actual evidence showing the necessity of involving an interprofessional team to prevent errors and adverse events, Wilson et al. also recommended an application to a more diverse clinical staff [16, 21]. The RoCAES-D can be carried out in German health care institutions without further restriction and hence, involve a larger number of clinical staff. Interventions that can be derived from performing these surveys will now be tailored more specifically and are therefore expected to be more effective as they address the whole multiprofessional team [30].

Limitations to our research concern the study population and the relatively low response rate as well as the model fit. In this survey, the response rate has been extremely low. However, the main purpose of this data collection was to gain a high variance and only use the data for the translation and validation process. For this, the sample size of *n* = 120, population structure and model fit are seen as appropriate [19, 22, 23]. To get an actual insight in the attitudes of health professionals, we recommend using the scale on institutional level. For this it is important to monitor the population structure and to achieve a higher response rate. On this basis, subgroup analysis, derivation of action plans or identification of needs for further investigation, e.g. employee interviews or process analyses, can be done properly. We also recommend a content-related revision of the scale after several years of use in different institutions. Possible additional cultural differences between the English, Chinese and German reporting culture have not been identified yet. The implementation of the scale for institutional use can help uncover cultural aspects and items that might need to be added for the German context. Even if the RoCAES-D showed a high reliability, the lower internal consistency on factor-level is reflected by the poor model fit of the modified model. This might support these cultural differences that require further investigation. Institutional usage with a data analysis on item-level, as well as additional psychometric testing to increase model fit is indicated in this regard.

The fact that a CFA had to be carried out instead of calculating divergent or convergent validity using a related survey underlines the existing research gaps. Nevertheless, translating and initially validating this scale might be a good starting point for further research and a progression for risk management in German health care institutions.

With 25 Items, the scale showed to be comprehensive. This comprehensiveness might explain the relatively low factor loadings. Yet, the purpose of this scale is to be an easy applicable screening device that identifies diverse starting points for further investigations or potential for further actions within a health care facility. As the scale is also short and concrete, we believe it can easily be embedded in a more extensive survey within institutions. It can for example be combined with a questionnaire addressing safety culture in general, or any other.

## Conclusions

In conclusion, the successful translation and initial validation of the RoCAES-D might be a good starting point for further research whereby data can be collected for further training or further investigations regarding an internal AE management system in clinics. Based on these results, a cultural adaptation of the scale needs to be done to initiate a large-scale usage of the questionnaire and to gain a useful instrument. Usage of the scale can contribute to improve patient safety on a long-term basis and area-wide through tailored interventions, such as education of employees or promoting a supporting reporting culture.

## Supplementary information

**Additional file 1.**

## Data Availability

The datasets used and analysed during the current study are available from the corresponding author on reasonable request. The German questionnaire can be seen in the appendix and is free for use when cited adequately.

## Abbreviations

AE: Adverse Events
CIRS: Clinical Incident Reporting System
RoCAES: Reporting of Clinical Adverse Events Scale
RoCAES-D: German Translation of the RoCAES
C-RoCAES: Chinese Translation of the RoCAES
CFA: Confirmatory Factor Analysis
EFA: Exploratory Factor Analysis
RMSEA: Root Mean Square Error of Approximation
SRMR: Standardized Root Mean Squared Residual
CFI: Bentler’s Comparative Fit Index
TLI: Tucker Lewis Index
AIC: Akaike’s Information Criterion
Df: Degrees of Freedom
