# Supplementary material for: German translation and validation of the Reporting of Clinical Adverse Events Scale (RoCAES-D)
Source: BMC Health Serv Res. 2020 Jul 25;20:689. doi: 10.1186/s12913-020-05546-2 (PMC7382079; doi:10.1186/s12913-020-05546-2)
Supplement: Supplementary file 1 — Additional file 1. [file 12913_2020_5546_MOESM1_ESM.docx]

**Skala der Berichterstattung von klinischen Zwischenfällen (RoCAES-D)**

Die unten aufgeführten Äußerungen wurden von anderen Fachkräften zur Beschreibung ihrer Sichtweisen bezüglich des Meldens von Zwischenfällen verwendet. Bitte lesen Sie jede Aussage. Kreuzen Sie die Felder der Skala so an, dass Ihre Meinung zur jeweiligen Aussage am besten wiedergegeben wird.

Als „Zwischenfall“ werden alle Aspekte des Gesundheitsversorgungsprozesses bezeichnet, die negative Auswirkungen für Patienten bedeuten könnten und nicht Teil des regulären Versorgungsprozesses oder des Krankheitsverlaufs sowie kein Resultat eigener Handlung sind.

A1. Waren Sie jemals Zeuge eines Zwischenfalls oder selbst in einen Zwischenfall involviert?

| □ | ja | □ | nein |
| --- | --- | --- | --- |

A2. Wenn ja, haben Sie jemals einen Zwischenfall gemeldet?

| □ | ja | □ | nein |
| --- | --- | --- | --- |

A3. Wie wahrscheinlich ist es, dass Sie zukünftig einen Zwischenfall melden?

| □ | sehr unwahrscheinlich | □ | unwahrscheinlich | □ | weder noch |
| --- | --- | --- | --- | --- | --- |
| □ | wahrscheinlich | □ | sehr wahrscheinlich |  |  |

|  |  | **Ich stimme voll und ganz zu** | **Ich stimme eher zu** | **Ich stimme eher nicht zu** | **Ich stimme überhaupt nicht zu** | **Keine Angabe** |
| --- | --- | --- | --- | --- | --- | --- |
| 1 | Das Melden von Zwischenfällen hilft dabei, Mitarbeiter zu identifizieren, die ergänzende Schulungen benötigen. | □ | □ | □ | □ | □ |
| 2 | Einen Zwischenfall zu melden oder nicht zu melden ist davon abhängig, wie viele Beteiligte von dem Zwischenfall wissen. | □ | □ | □ | □ | □ |
| 3 | Es liegt nicht in meiner Verantwortlichkeit, Zwischenfälle von Kollegen zu melden. | □ | □ | □ | □ | □ |
| 4 | Das Melden von Zwischenfällen hilft, Patienten zu schützen. | □ | □ | □ | □ | □ |
| 5 | Das Melden von Zwischenfällen gibt Anderen die Möglichkeit, mich zu überprüfen. | □ | □ | □ | □ | □ |
| 6 | So lange meine Kollegen von den Ereignissen lernen, besteht keine Notwendigkeit, diese zu melden. | □ | □ | □ | □ | □ |
| 7 | Das Melden von Zwischenfällen wirkt sich negativ auf die Karriere des Mitarbeiters aus, welcher den Fehler gemeldet hat. | □ | □ | □ | □ | □ |
| 8 | Das Vorgehen der Berichterstattung ist in dieser Klinik eindeutig. | □ | □ | □ | □ | □ |
| 9 | Ich führe meinen Beruf nicht ordnungsgemäß aus, solang ich Zwischenfälle nicht melde. | □ | □ | □ | □ | □ |
| 10 | Kleinere Zwischenfälle sollten nicht gemeldet werden. | □ | □ | □ | □ | □ |
| 11 | Meine Kollegen erwarten von mir, dass ich Zwischenfälle melde. | □ | □ | □ | □ | □ |
| 12 | Wenn ich einen Zwischenfall melde, bringt mich dies in Schwierigkeiten. | □ | □ | □ | □ | □ |
| 13 | Aus Handlungsanweisungen der Klinik geht klar hervor, welche Art von Zwischenfällen gemeldet werden sollten. | □ | □ | □ | □ | □ |
| 14 | Ausschließlich seltene Zwischenfälle sollten gemeldet werden. | □ | □ | □ | □ | □ |
| 15 | Einen Zwischenfall in der Akte des Patienten zu dokumentieren ist genauso effektiv wie das Ausfüllen eines Meldeformulars. | □ | □ | □ | □ | □ |
| 16 | Bestärkendes Verhalten von Vorgesetzten motiviert mich, Zwischenfälle zu melden. | □ | □ | □ | □ | □ |
| 17 | Ein in der Klinik etabliertes Berichterstattungssystem ermutigt Mitarbeiter, Zwischenfälle zu melden. | □ | □ | □ | □ | □ |
| 18 | Das Melden von Zwischenfällen lässt alle anderen wissen, dass ich einen Fehler gemacht habe. | □ | □ | □ | □ | □ |
| 19 | Es ist mir nicht erlaubt, Zwischenfälle zu melden. | □ | □ | □ | □ | □ |
| 20 | Man sollte nur die Zwischenfälle melden, aus denen eine Lehre gezogen werden kann. | □ | □ | □ | □ | □ |
| 21 | Das Melden von Fehlern ist eine Methode, um jemanden gezielt zu beschuldigen. | □ | □ | □ | □ | □ |
| 22 | Fehler können nicht verhindert werden. Daher gibt es keinen Grund, Zwischenfälle zu melden. | □ | □ | □ | □ | □ |
| 23 | Das Melden eines Zwischenfalls bringt meine Kollegen dazu, über meinen Schuldanteil zu spekulieren. | □ | □ | □ | □ | □ |
| 24 | Wenn Zwischenfälle gemeldet werden, müssen sich Personen für ihr Handeln verantworten. | □ | □ | □ | □ | □ |
| 25 | Kollegen reagieren häufig unbesorgt, wenn Zwischenfälle passieren. | □ | □ | □ | □ | □ |
